# Self-sustainable autonomous soft actuators

**DOI:** 10.1038/s42004-024-01142-1

**Published:** 2024-03-19

**Authors:** Zhen-Zhou Nie, Meng Wang, Hong Yang

**Affiliations:** School of Chemistry and Chemical Engineering, State Key Laboratory of Digital Medical Engineering, Institute of Advanced Materials, Southeast University, Nanjing, 211189 China

**Keywords:** Soft materials, Polymer chemistry, Molecular machines and motors, Polymers

## Abstract

Self-sustainable autonomous locomotion is a non-equilibrium phenomenon and an advanced intelligence of soft-bodied organisms that exhibit the abilities of perception, feedback, decision-making, and self-sustainment. However, artificial self-sustaining architectures are often derived from algorithms and onboard modules of soft robots, resulting in complex fabrication, limited mobility, and low sensitivity. Self-sustainable autonomous soft actuators have emerged as naturally evolving systems that do not require human intervention. With shape-morphing materials integrating in their structural design, soft actuators can direct autonomous responses to complex environmental changes and achieve robust self-sustaining motions under sustained stimulation. This perspective article discusses the recent advances in self-sustainable autonomous soft actuators. Specifically, shape-morphing materials, motion characteristics, built-in negative feedback loops, and constant stimulus response patterns used in autonomous systems are summarized. Artificial self-sustaining autonomous concepts, modes, and deformation-induced functional applications of soft actuators are described. The current challenges and future opportunities for self-sustainable actuation systems are also discussed.

## Abstract (web summary)

Self-sustainable autonomous soft actuators have emerged as naturally evolving out-of-equilibrium systems that do not require human intervention. Here, the authors discuss recent advances in the field, with a focus on shape-morphing materials, motion characteristics, built-in negative feedback loops, and constant stimulus response patterns.

## Introduction

Self-sustainable autonomous locomotion is an out-of-equilibrium phenomenon and an intelligent behavioral characteristic of soft-bodied living organisms^1–4^. Biological systems of varying lengths and complexities have excellent survival, foraging, and shape-shifting camouflage functions for interacting with a range of environments^5,6^. Soft robots made from soft materials hold the promise for tapping the intelligence found in nature. Hence, they have attracted considerable attention in the exploration of artificial self-sustainable autonomous systems that can greatly advance human-machine interaction in agriculture, industry, biomedicine, and other fields^2,7^. The current design of automated soft robots is mainly derived from algorithms and software that also issue instructions to soft modular components for executing specific tasks, such as in soft-robotic arms^8–10^. Deep learning is another intelligent technology that is used to construct environmentally adaptive, autonomous, sustainable robotic systems^11^. This enables micro-swarms to make autonomous decisions without human intervention as they navigate a wide range of unstructured environments.

Soft actuators have different mechanical design principles than those discussed above, and can couple autonomous responses under complex environmental changes and self-sustaining motions under continuous stimulation, thereby becoming a new category of naturally evolving drive systems^12–19^. In soft actuators, smart materials such as liquid crystal elastomers (LCEs) and hydrogels are stimulated by external inputs such as heat, light, and moisture to achieve shape deformation^20–27^. Structural morphologies and internal molecular arrangements serve as “programming tools” that can confer motion patterns and actuation functions to soft actuators^28–30^. More essentially, built-in negative feedback loops are used to construct nonequilibrium actuator systems that transition between multiple dynamically stable and metastable states under constant stimulation^1,31–34^. These feedback loops are established by cooperating with appropriate stimulus sources and deformation patterns. It is worth noting that without a built-in loop, stimulus-triggered actuators may reach their deformation limits and remain permanent in that state^35–43^. Therefore, reversible shape transformation requires a manual on/off stimulation intervention, which is complex and time-consuming. In contrast, in self-sustaining autonomous mechanisms, soft actuators directly sense a constant stimulus, deform to a position away from the stimulus source, and autonomously return to their initial state based on an embedded negative feedback loop^44,45^. This simple strategy greatly reduces the need for mechanical modules, such as sensors, controllers, batteries, and drive motors, thereby improving the sensitivity, responsiveness, portability, and safety of soft robots in human-machine or environment-machine interactions. Therefore, self-sustaining autonomous mechanisms have significant potential for use in unprecedented biomimetic materials, miniaturized autonomous robots, unstructured terrain surveying, intelligent manufacturing, and physical intelligence^46^ (i.e., physically encoding sensing, actuation, control, logic and decision-making into soft robotic bodies).

**Fig. 1:**
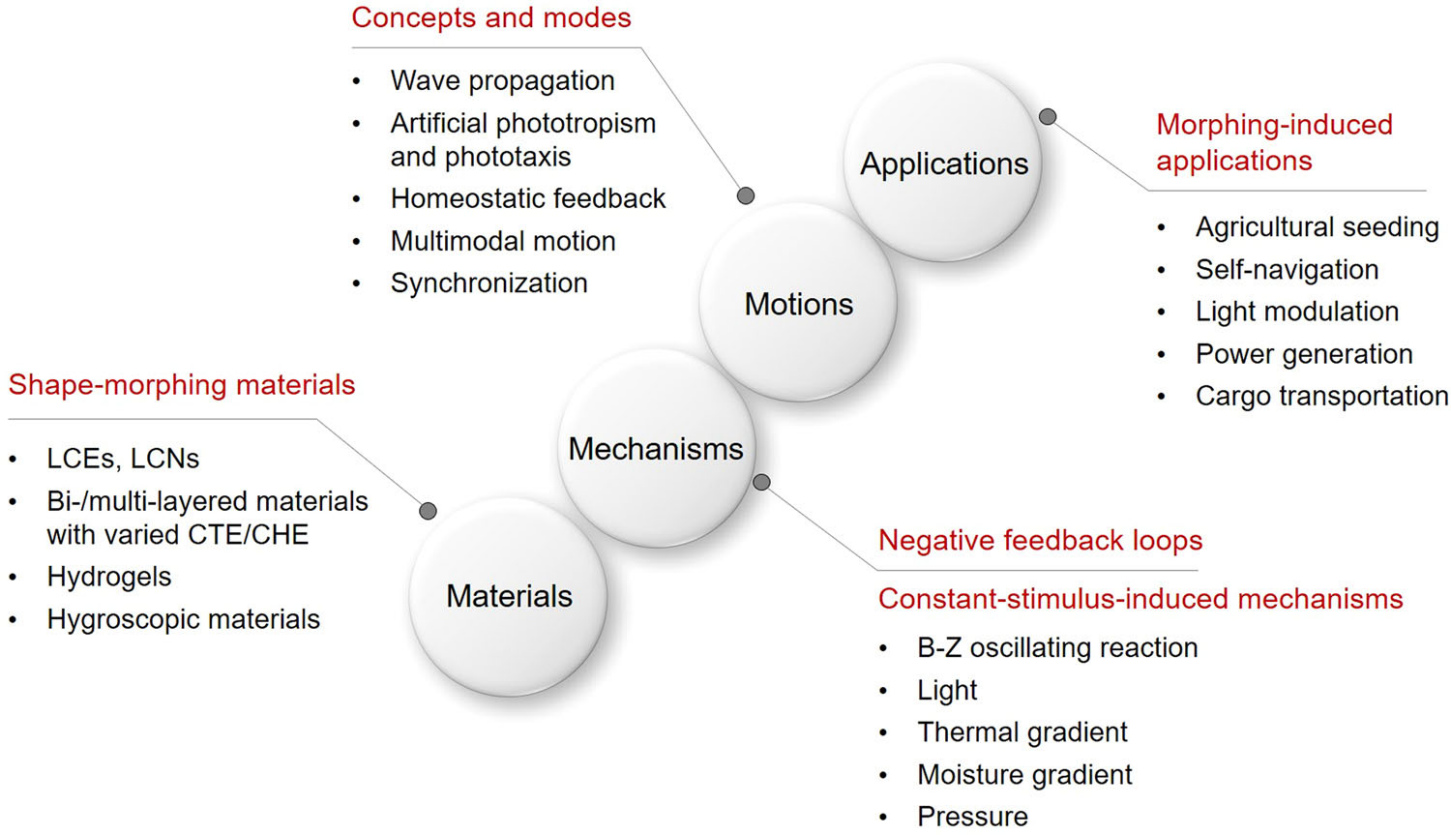
A general roadmap toward self-sustainable autonomous soft actuators. Recent advances in self-sustainable autonomous soft actuators include shape-morphing materials, mechanisms based on negative feedback loops and constant stimuli, concepts and motion modes, and morphing-induced applications.

In this perspective article, we present an overview of the recent advances in self-sustainable autonomous soft actuators (Fig. 1). Stimulus-responsive shape-morphing materials and their actuation principles for self-sustained locomotions are introduced in “Shape-morphing materials used for self-sustainable motions” section. Negative feedback loops embedded in soft actuators and five constant-stimulus-induced self-sustaining mechanisms are described in “Constant stimuli-induced self-sustaining mechanisms” section. Self-sustainable autonomy concepts and motion modes are discussed in “Self-sustainable autonomous concepts and motion modes” section. Self-sustainable applications of soft actuators are described in “Autonomous, sustainable morphing-induced applications” section. Finally, we present our views on the current challenges and future opportunities in the “Summary and outlook” section.

### Shape-morphing materials used for self-sustainable motions

**Fig. 2:**
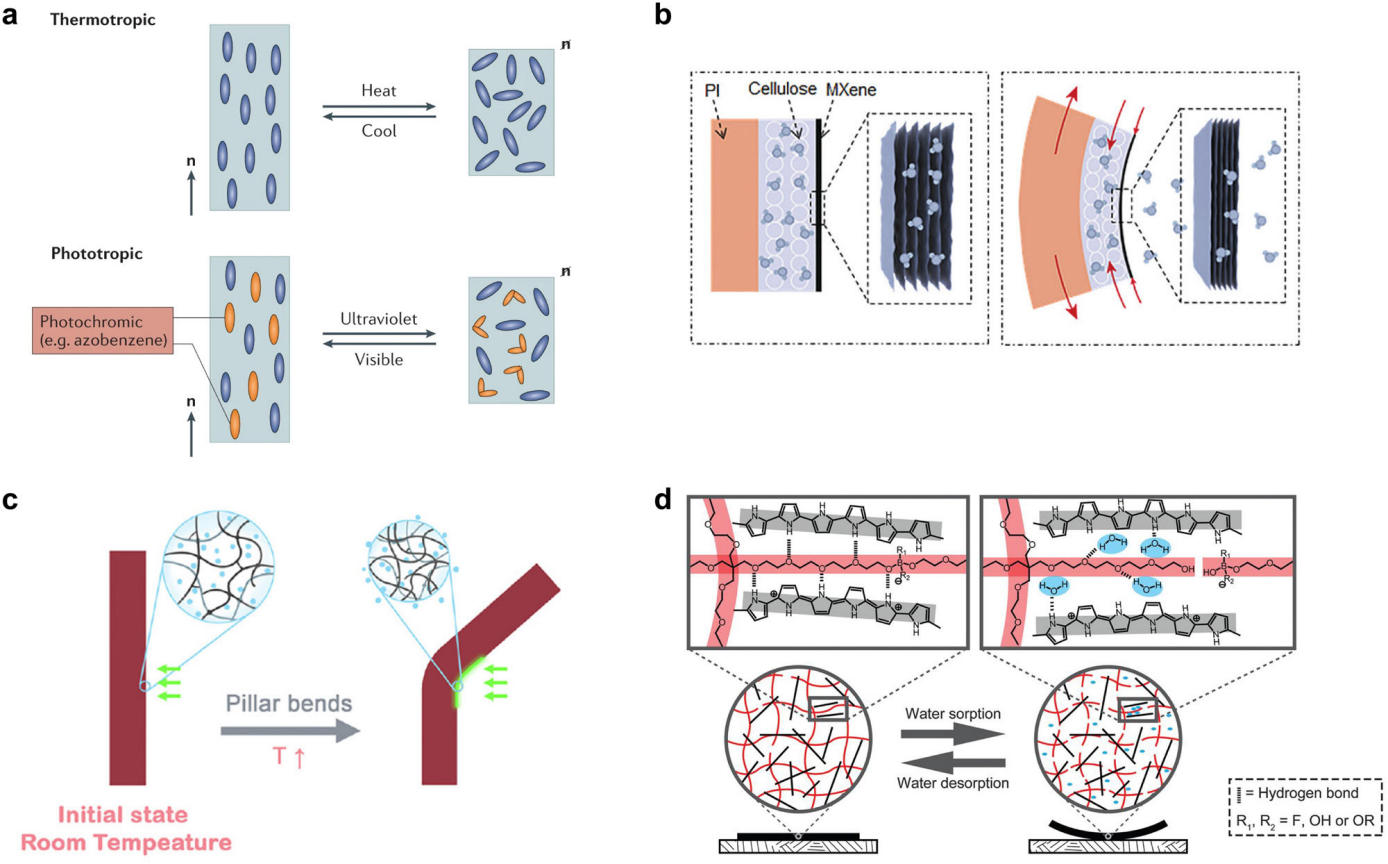
Shape-morphing materials used for self-sustainable motions. **a** LCEs and LCNs. Reprinted with permission from Ref. ^51^, Springer Nature. **b** Bi-layered materials with varied coefficient of thermal expansion. Reprinted with permission from Ref. ^57^, WILEY-VCH. **c** Hydrogels. Reprinted with permission from Ref. ^69^, AAAS. **d** Hygroscopic materials based on swelling-deswelling transitions. Reprinted with permission from Ref. ^70^, AAAS.

Traditional soft robots comprise soft materials, rigid motors, controllers, and sensors. Unfortunately, most of the well-developed motors and other components are not transferable to soft bodies^47,48^. To address this issue, stimulus-responsive shape-morphing materials have been demonstrated to be excellent candidates for reinventing motors and sensors for soft moving bodies^49,50^. As shown in Fig. 2, the shape-morphing materials used for self-sustainable autonomous actuator systems mainly include LCEs and liquid crystal networks (LCNs), bi-/multi-layered materials with various coefficients of thermal expansion (CTE), and hygroscopic expansion (CHE), hydrogels, and other hygroscopic materials based on swelling-deswelling transitions.

LCEs are moderately crosslinked polymer networks consisting of liquid crystalline (LC) mesogens, flexible spacers, and a small amount of crosslinker^51–54^. As a result, LCEs have soft elasticity (modulus: kPa−MPa) and their glass transition temperatures *T*_g_ are usually below room temperature. The actuation mechanism of LCEs is derived from a stimuli-triggered transition from the LC phase to an isotropic phase. Figure 2a presents the widely used thermotropic LCEs as an example. Above the clearing point temperature, *T*_i_, the orientation of the LC mesogens and the polymeric chain configuration undergoes dimensional change, causing obvious macroscopic shape morphing. As the temperature decreases, the LC orientation and chain configuration recover, resulting in the expansion of the LCE to its initial size. The mechanical response of LCEs is reversible, and the corresponding actuation strain, which ranges from 20% to 81%, exceeds that of human skeletal muscles (20−40%). Photothermal LCEs exhibit thermotropic morphing responses. LCNs are the highly crosslinked glassy polymers (modulus *E*: MPa−GPa) fabricated by polymerization of high concentrations of multifunctional liquid crystalline monomers^55,56^. Consequently, LCNs exhibit a limited shape change of 5% or less. The stimulus response of LCNs is generally based on the phototropic mechanism of *trans*-to-*cis* isomerization of azobenzene. Under UV irradiation, the azobenzene moieties of the cross-linked network undergo photo-isomerization from rod-shaped *trans* to curved *cis*, and the length of azobenzene moieties decreases from approximately 9.0 Å to 5.5 Å. Molecular motion can disrupt the orientation of liquid crystals and induce the ordered-disordered phase transitions. Because of the high molar extinction coefficient of azobenzene at 365 nm, a local volume contraction on the exposed surface is realized, which triggers macroscopic bending of the LCN materials. Sensitive, reversible, and multi-stimulus-responsive properties enable LC polymer-based materials to be perfectly suitable for constructing self-sustainable autonomous systems.

Bi-layered materials with varying CTE are also thermoresponsive shape-morphing materials^57–59^. Many materials expand upon heating and shrink upon cooling, indicating a positive CTE, such as polyethylene (PE) polymer tape (Longitudinal axis: 502 × 10^−6^ °C^−1^; Short axis: 91.1 × 10^−6^ °C^−1^ at 40 °C)^59^. Two or more material films with different CTE can be bonded at the interface to fabricate bilayer or multilayer actuators. Under heating conditions, the bi-layered materials generate an asymmetric force and demonstrate bending deformation towards the side with a small CTE because the expansion of the side with a high CTE is larger than that of the side with a small CTE. After removing the heat stimulus, the bilayered strip returned to its original state. Additionally, humidity-responsive materials with high CHE such as cellulose and MXene (Ti_3_C_2_T_x_) comprising some hydrophilic groups such as hydroxyl, carboxyl or pyrrolyl, can also be used for building bi-/multi-layered shape-morphing materials^45^. As indicated in Fig. 2b, the trilayered strip contains a photothermal MXene layer, a cellulose layer, and a polyimide (PI) strip^57^. Under light irradiation, the PI film with a high CTE expands along the longitudinal direction, whereas the MXene-cellulose film realizes thermal contraction due to the desorption of water molecules. This synergistic effect promotes asymmetric mechanical deformation in the MXene-based composite film. After removing the light, the hygroscopic characteristics of the MXene-cellulose layer and contraction of PI transform from a curved to a flat actuator morphology.

Hydrogels are highly hydrophilic crosslinked polymeric networks with high water content^60–62^. The hydrophilic groups in polymer chains, such as amino and hydroxyl groups, determine their water-absorbing properties. The crosslinking networks counteract the affinity for water, resulting in the retention of polymeric structures by preventing them from dissolution^63–66^. The mechanical response of hydrogels is derived from swelling and deswelling deformations, which are generally affected by changes in hydrophilicity or temperature. The Belousov-Zhabotinsky (B-Z) reaction is a well-known classic oscillating chemical reaction^67^. In self-oscillating hydrogels, the redox changes in the catalyst moiety (Ru(bpy)_3_^2+^ ↔ Ru(bpy)_3_^3+^) in polymer chains can adjust swelling ratio and even volume phase transition temperature of hydrogels. This is because the hydrophilicity of the network increases in the oxidized Ru(III) state and drops in the reduced Ru(II) state^68^. As shown in Fig. 2c, He et al. developed visible photothermally responsive hydrogel systems with arbitrary directional movements in fluids and high damping coefficients^69^. The gold nanoparticles embedded hydrogel (poly(N-isopropylacrylamide)) could achieve 70% of volumetric change at 32 °C, which is a low actuation temperature and therefore beneficial for rapid morphing response. Under light illumination, the hydrogel pillars were curved owing to local volumetric contraction. When the local responsive region could not receive photonic energy, the hydrogel network swelled. Consequently, no volumetric difference remained between the sides of the pillar, resulting in reversible motions.

In addition to hydrogels, other hygroscopic materials are used for self-sustained actuation, including inorganic materials (e.g., MXene^57^ and carbon materials^20^) and organic polymers (e.g., hybrid polypyrrole and polyol-borate materials, woody materials^70^). The hydrophilic groups and ions in these hygroscopic materials interact with water molecules, causing macroscopic swelling and bending of these materials (Fig. 2d). Subsequently, the local swelling region moves away from the humidity source^70^. Consequently, the internal water molecules can be removed from the materials and actuators, completing the reversible shape transformation. Hydrogels and hygroscopic materials differ in two ways. Hydrogels are present in aqueous media, whereas hygroscopic materials are usually present in air. In addition, hydrogels tend to deswell locally and contract upon exposure to external light or heat. However, hygroscopic materials are stimulated by humidity and swell to induce a bending motion in the direction opposite to that of the humidity source.

### Constant stimuli-induced self-sustaining mechanisms

Biological systems can harness energy directly from a constant ambient environment and perform self-sustainable autonomous motions for environmental adaptation and task execution. Autonomous behaviors mainly include periodic oscillations (e.g., tail swing, heartbeats, and neuron impulses) and continuous locomotion (e.g., tumbling, rolling, rotation)^30^. Biologically sustainable features are out-of-equilibrium phenomena based on built-in negative feedback loops that are transformations between two or more kinetically stable and metastable states^1,69^.

**Fig. 3:**
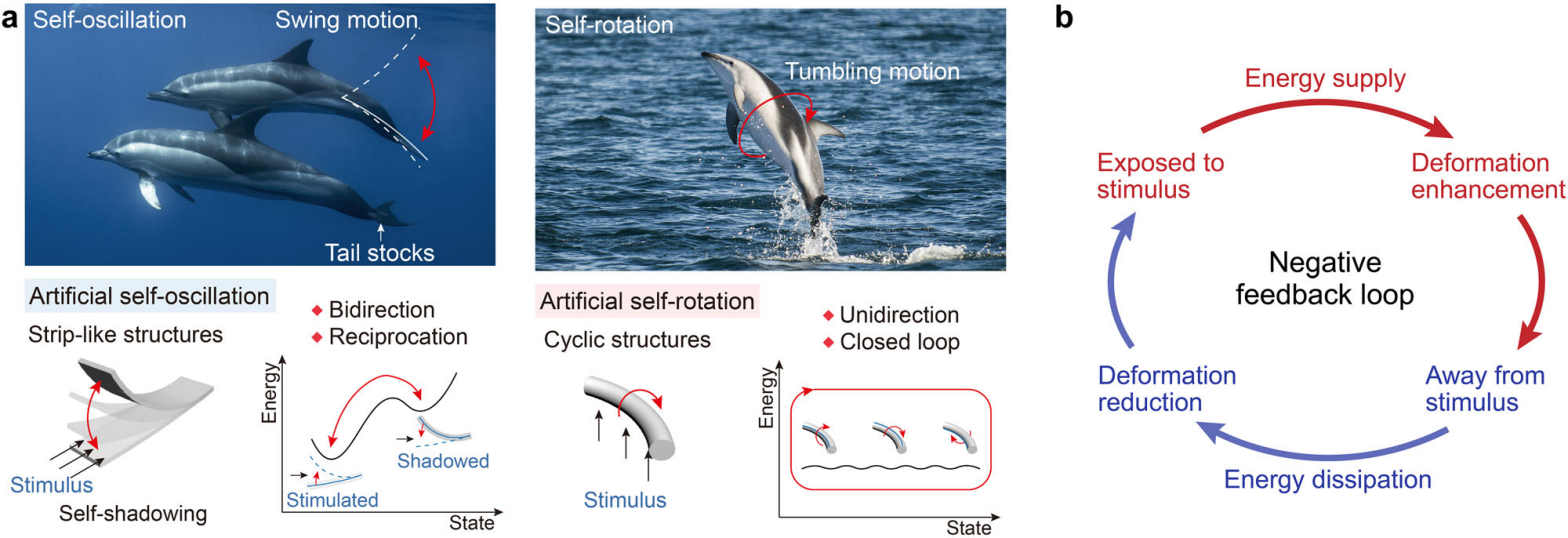
Self-sustainable autonomous motion mechanism of soft actuators. **a** Self-oscillation and self-rotation of biological systems and soft actuators. Reprinted with permission from Ref. ^30^, WILEY-VCH. **b** Negative feedback loop of self-sustainable autonomous motions.

Artificial soft actuators can build lifelike feedback mechanisms utilizing energy supplied by environmental sources. As shown in Fig. 3a, self-sustained oscillations can interconvert between the stable and metastable states of soft actuators, which generally rely on strip-like structures with self-shadowing effects^71–73^. For continuous self-rotation, the actuating energy generated by the actuators can overcome all the states, resulting in motion loops and endless cycles^74,75^. Consequently, the conversion of all the states is unidirectional and usually requires continuous cyclic actuator structures. Regardless of the type of self-sustained motion, out-of-equilibrium phenomena are key factors that depend on the negative feedback loop and energy dissipation. Taking self-oscillation as an example, the strip-like oscillator is initially exposed to stimuli such as light to obtain the energy supply (Fig. 3b). Because of their shape-morphing features, the accumulated energies in soft actuators can be transformed into mechanical energies for deformation enhancement. However, intense locomotion causes the local response regions of the oscillator to move away from the stimulus source and thereby interrupting the energy supply. Subsequently, the energy consumption and dissipation contributed to a reduction in the deformation strain. Eventually, the soft oscillator can recover its original state, receive energy supply again, and enter the next motion cycle. A negative feedback loop enables robust out-of-equilibrium phenomena and self-sustainable systems.

**Fig. 4:**
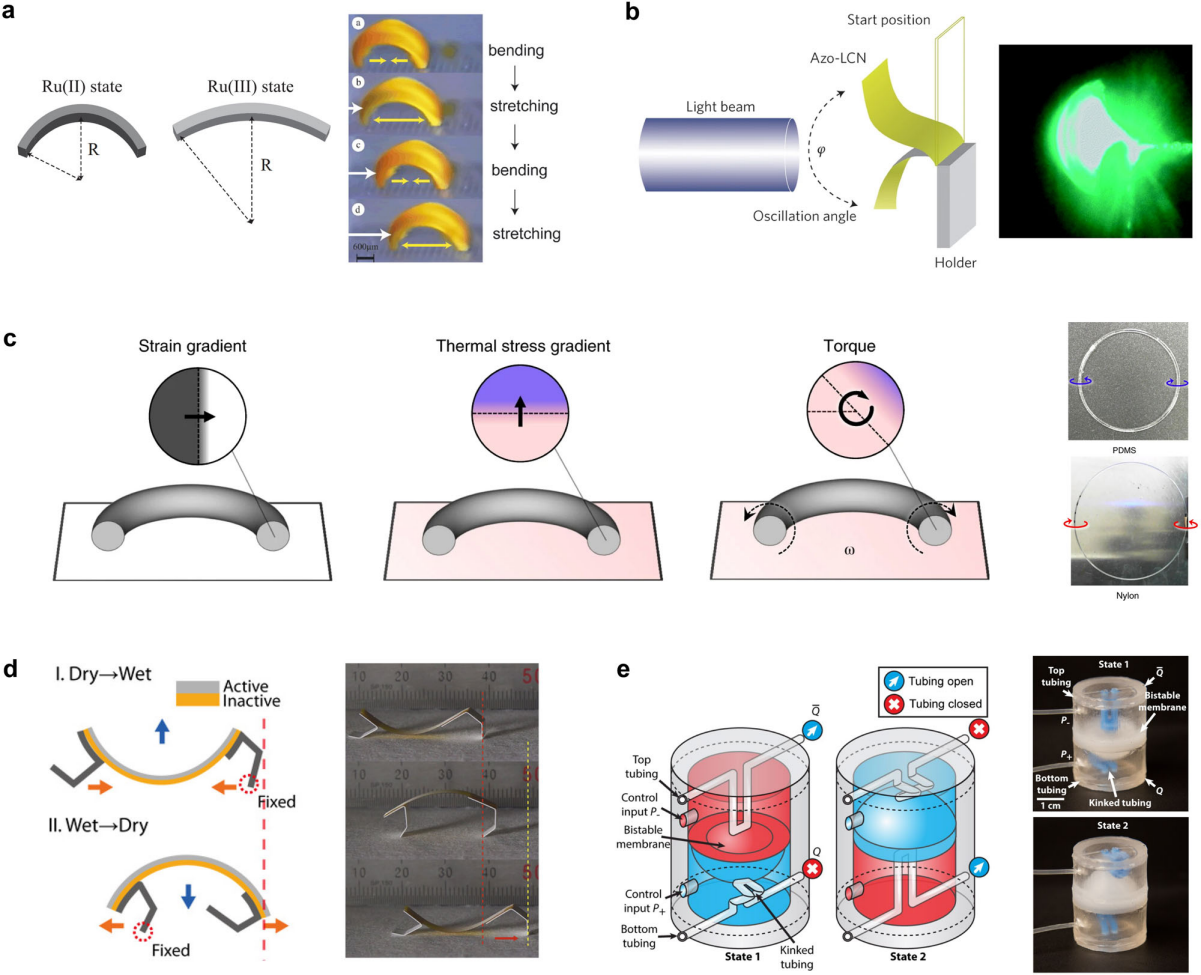
Multiple constant stimuli-induced self-sustainable autonomous actuators. Self-sustainable autonomous actuators under constant (**a**) B-Z oscillating chemical reaction. Reprinted with permission from Ref. ^19^, WILEY-VCH. **b** Light irradiation. Reprinted with permission from Ref. ^78^, RSC. **c** Thermal gradient. Reprinted with permission from Ref. ^75^, Springer Nature. **d** Moisture gradient. Reprinted with permission from Ref. ^81^, AAAS and (**e**) Pressure. Reprinted with permission from Ref. ^18^, AAAS.

Self-sustainable autonomous motion is driven by multiple constant stimuli, including the B-Z oscillating chemical reaction, light, thermal gradient, moisture gradient, and pressure, as shown in Fig. 4. The shape-morphing material selection, actuation mode design, and ingenious response principles for different stimuli are discussed below. As shown in Fig. 4a, under constant chemical conditions and temperatures, the B-Z oscillation reaction can occur inside the hydrogels to enable energy conversion from autonomous chemical oscillation to mechanical oscillation^19^. Ruthenium(II) tris(2,2’-bipyridine) (Ru(bpy)_3_^2+^) is a catalyst for the B-Z oscillatory reaction and is covalently bonded to polymeric chains. In the aqueous solution of the B-Z reaction, except for the catalyst, the hydrophilic changes in the hydrogel-based actuator were realized by the periodic redox changes in the catalyst in the gels, resulting in a swelling-deswelling transformation. Because of the two different surfaces of the plates during the polymerization process, the gel actuator had a gradient content distribution for each component. The hydrophilic side had a higher swelling ratio than the hydrophobic Ru(bpy)_3_^2+^ side, leading to the actuator bending towards the hydrophobic side and implementing directional locomotion. Under constant conditions, the Ru–(bpy)_3_ group was oxidized and reduced by the B-Z reaction, which triggered periodic self-oscillations and walking. This is the first example of embedding B-Z oscillatory reaction into soft hydrogel actuators to achieve autonomous locomotions^19^. Other chemical-to-mechanical mechanisms include non-redox reactions^76^, pH-controllable mechanisms^77^, and chemo-mechano-chemical autonomous regulation^17^.

Light-driven self-sustainable systems have been widely developed in recent years because light is non-invasive, remotely controllable, and directly obtained from nature. Autonomous locomotion under constant light illumination is derived from self-shadowing effects, which are typically realized by adjusting the light direction. The conversion of light energy to mechanical work in artificial actuator systems is mainly divided into two categories: photochemical switching between *trans*-*cis* isomers^31,33,34,78^ and photothermal effects^32,36,73^. As demonstrated in Fig. 4b, Bunning et al. first reported fast and large light-driven oscillations of azo-LCN cantilever actuators^31,78^. The light beam was directed perpendicular to the LCN cantilever. The incident Ar^+^ laser aligned the mesogens of the LCN strip and drove the cantilever tip. The back surface of the actuator was initially exposed, and a downstroke was achieved. In this state, light could irradiate the front surface and bend upward, causing a sequential feedback loop and high performance (oscillatory frequency: 30 Hz; amplitude: >170°). Notably, the LCN cantilever can be activated by sunlight and exhibits a high fatigue resistance over 250,000 motion cycles. Photothermally driven strip-like actuators also exhibit self-shadowing and thermal actuation. Soft actuators are generally coated or embedded with dyes having photothermal conversion capabilities, such as carbon materials, gold nanoparticles, and organic molecules^79^. Under constant light irradiation, the photothermal-mechanical energy conversion induces a feedback loop between the upstroke and downstroke of the autonomous actuators.

Heat energy is an untethered and readily available stimulus. Hence, thermally responsive actuators can rely on a heating input for autonomous motility. The thermal gradient is produced by placing the actuator on a hot stage, which generates thermo-mechano-feedback for a continuous response^80^. As shown in Fig. 4c, the polymeric rod is closed into a circular loop, in which the inside and outside regions undergo longitudinal compression and tension, respectively^75^. Consequently, the circular actuator exhibits a topological pre-strain, and the internal structural symmetry is broken. The temperature gradient between the hot plate and ambient air triggers a heating strain difference in the circular actuator along the direction perpendicular to the ground. This direction is normal to the strain gradient of the rod, which results in torque around the axis of the rod, further inducing continuous internal self-sustained rotation. The actuators based on polydimethylsiloxane (PDMS) with a positive CTE and nylon with a negative CTE rotate along the outward and inward directions, respectively. Essentially, the geometric zero-energy modes are proposed to elicit rotary motion in elastic materials without the need for rigid wheels to travel around an axis^75^. Moreover, some spiral and fibrous actuators are capable of self-rolling through non-uniform actuation strains under thermal gradients.

Natural and artificial hygroexpansive actuators are gaining popularity because of their potential to capture energy from changes in environmental humidity. When soft actuators are placed near a moist surface, a humidity gradient exists along the direction perpendicular to the surface^81^. The corresponding gradient can induce a strain gradient in the thickness direction of the actuators for curved morphing. In this state, the local responsive region in soft materials is far away from the wet substrate and then deswells in a reversible, self-sustainable loop. Most self-locomotive actuators exhibit unidirectional bending and rolling. As shown in Fig. 4d, a hygroscopically driven bilayered actuator can self-walk in a ratcheted fashion under a constant humidity gradient to rectify the movement direction. The aligned nanofiber-based actuator is fabricated by directional electrospinning, which is beneficial for rapid swelling-deswelling transition along the longitudinal direction. With optimal ratchets and a mathematical analysis of the actuation, the actuators realize directional oscillating bending movements at high speeds.

In addition to common environmental stimuli, a constant pressure can be used to construct an autonomous feedback loop and control engineering robotics. Figure 4e shows that the bistable elastomeric valve is composed of a hemispherical membrane for the separation of the two chambers^18^. The pressure difference between the bottom *P*_+_ and top *P*_-_ chambers can induce the shape-morphing of the soft membrane. In State 1, the airflow passed through the upward tube, and the pressure increased in the top chamber increased. Airflow was blocked through the bottom chamber by squeezing and twisting the tubes. Under a constant pressure (constant input), the pressure difference between the two chambers can reach a threshold and trigger an upward snap-through motion of the soft valve. In State 2, the upward tube was squeezed to block the air input. However, the air inside the cavity flowed out. The soft membrane then snapped downward and returned to its initial state. This strategy is further developed for autonomous grippers and self-sustaining earthworm-like movements.

### Self-sustainable autonomous concepts and motion modes

The underlying mechanism of self-sustainable autonomous actuators is inspired by the out-of-equilibrium and dissipative phenomena in living organisms. Even more fascinating is the fact that biological systems are rich in complex behavioral mechanisms and physical intelligence. Scientists constantly explore and imitate these biological features in attempting to make artificial actuator systems come to “life.” Consequently, this chapter discusses the design and construction of these biomimetic concepts and the motion modes of self-sustainable autonomous systems.

**Fig. 5:**
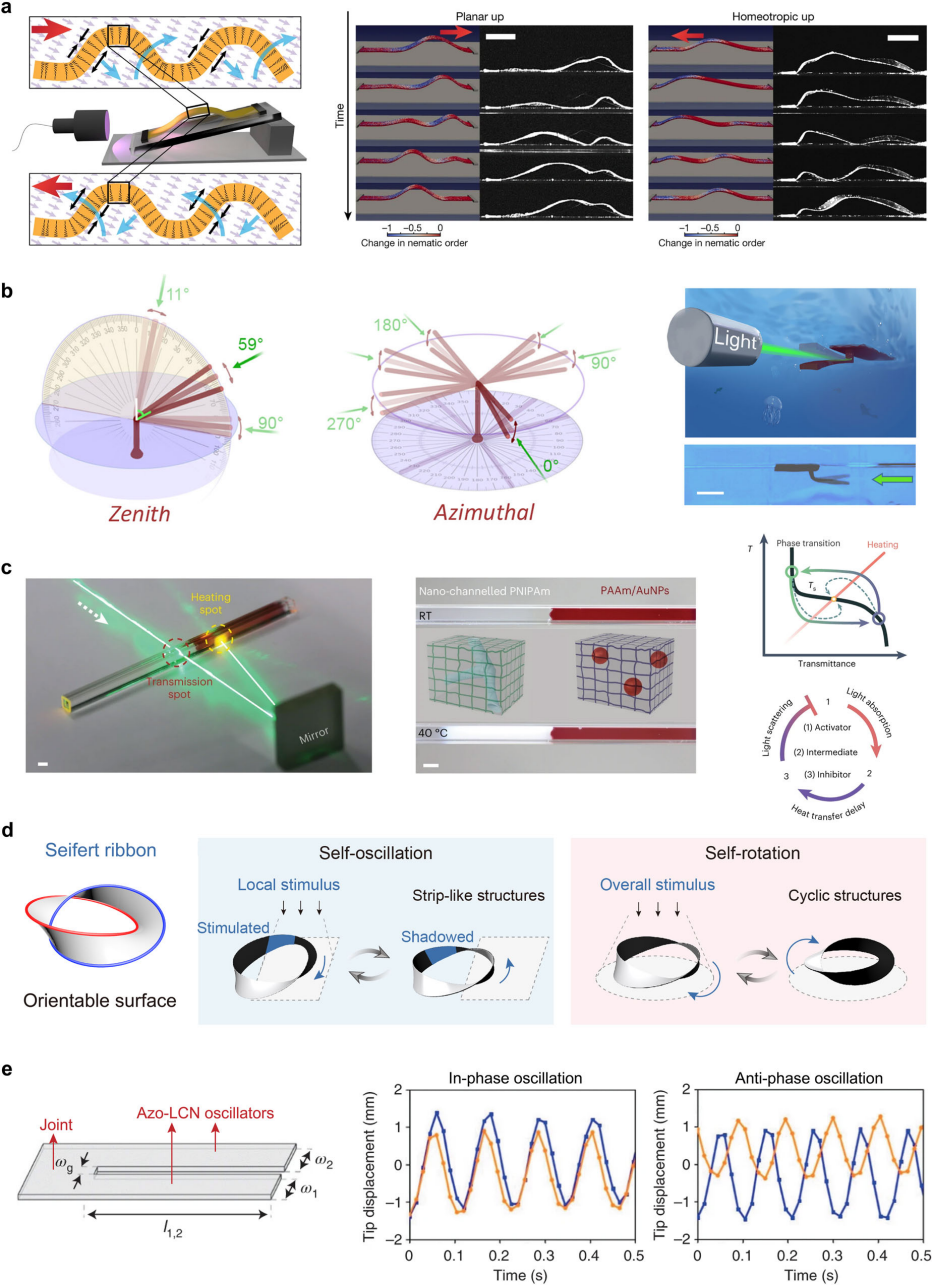
Self-sustainable autonomous concepts and motion modes. **a** Self-sustainable wave propagation of photoactive azo-LCN film. Reprinted with permission from Ref. ^82^, Springer Nature. **b** Artificial phototropism and phototaxis of underwater actuator. Reprinted with permission from Ref. ^69^, AAAS. **c** Homeostatic feedback-controlled oscillations. Reprinted with permission from Ref. ^88^, Springer Nature. **d** Multimodal self-sustainable autonomous Seifert ribbon actuator. Reprinted with permission from Ref. ^30^, WILEY-VCH. **e** Synchronization and collective motion of coupled azo-LCN oscillators. Reprinted with permission from Ref. ^89^, Springer Nature.

Spontaneous mechanical waves without human intervention are shown in Fig. 5a. Broer et al. embedded azo derivatives with rapid *cis*-to-*trans* thermal relaxation into LCNs, which consequently accelerated the mechanical response of wave-like actuators^82^. This splay-aligned configuration of the LCNs contributed to the large amplitude of shape morphing. Under constant light illumination, waves were generated owing to a feedback loop driven by the self-regulated shadowing effect. Autonomous wave motion was further developed to enable the rejection of contaminants and ensure self-propelled transport. Furthermore, a carbon nanotube/PDMS-based bilayer film produced human-inspired sit-up motion under constant white light illumination^72^. Phototaxis continuous wave-like self-oscillation was further applied to the crawling locomotion and energy-harvesting devices.

Phototropism is a phenomenon in which plants grow and orient themselves towards solar energy to maximize energy capture^83^. Phototaxis is another phenomenon in which some microorganisms and animals are capable of moving towards or away from light sources to prey on and forage for food. He et al. presented ingenious artificial phototropism and phototaxis concepts for self-locomotive actuators^69,84,85^. A remotely constant light-fueled omnidirectional oscillation is shown in Fig. 5b. Owing to the high degrees of freedom of the hydrogel pillars, the light direction can precisely steer directional self-oscillatory motions in the entire three-dimensional space^56^. Furthermore, an autonomous phototaxis swimmer generated sufficient shaking force to overcome aqueous damping and self-propel away from the beam. As a result, these photo-responsive hydrogel pillars can be conveniently tuned for artificial phototropic orientation and phototactic movement in fluid media.^69^ Qian et al. reported the full-space phototaxis of a soft underwater vehicle based on self-regulation and the directional flow around itself^86^. Natural sunlight drives self-oscillating locomotion directly in a fluid environment. Moreover, natural sunlight fluctuations could replace the on/off stimulation to promote self-regulation and autonomous locomotion of inchworm-like soft robotics^87^.

Novel artificial dissipative systems for homeostatic thermal oscillations are shown in Fig. 5c. The encapsulated composite hydrogel is composed of thermally responsive PNIPAm and light-absorbing PAAm/AuNP gels with photothermal abilities^88^. Under constant light irradiation, light can transmit the PNIPAm components and be reflected in the PAAm/AuNP gels, which convert photonic energy to thermal energy. The increase in temperature causes the phase transition and opacification of PNIPAm, and the gels cannot receive photons; thus, the temperature would decrease. Consequently, a photo-fueled negative feedback loop with a thermally coupled delay was built into the gel system for temperature oscillation. The LCE fin arrays were attached to the gel actuators for the gel-LCE assembly. Derived from thermal oscillations, LCEs realize synchronized but nonidentical shape morphing and cargo transportation.

Our group developed the multimodal self-sustained movements of photoresponsive LCE-based Seifert ribbon actuators (Fig. 5d). Because of the oriented surface of the Seifert ribbon, the exposed and shadowed regions of the topological actuators would persistently harness and shield light under constant light stimulation, respectively^30^. The generated twisted energy can be spread across the surface to enable self-sustaining movement. A topological soft actuator can autonomously perceive changes in the beam region, and the responsive component becomes either a discontinuous strip-like structure or continuous toroidal structure. Eventually, life-like adaptive switches between self-sustained oscillatory and rotary locomotion were implemented. Bistable structures are utilized to construct self-sustained systems that can endow soft actuators with snap-through functions rather than uniform motions^36,77^. Priimagi et al. designed optically driven freestyle self-oscillators^73^. By regulating the non-periodic light beam position, the LCN oscillators performed various periodic deformations, including reversible bending, twisting, and contracting-expanding motions. Interestingly, changes in temperature and humidity also trigger multimodal self-sustainable locomotions^14,86^.

Synchronization and collective motion are the fascinating phenomena of self-organization in nature and are crucial for communication and coupled actuation of self-sustainable actuators. As shown in Fig. 5e, the synchronization of azo-LCN actuators under constant light illumination was inspired by Huygens’ synchrony^89^. Two oscillatory films could interact with each other’s motion by coupling in-phase and anti-phase oscillations at steady state. Synchronization was determined by the mechanical coupling in the joint of the film actuators, which was further adjusted by the stiffness and damping of the joint elements.

### Autonomous, sustainable morphing-induced applications

Stimulus-responsive self-sustainable actuators without external control have unique advantages in the construction of intelligent devices. The self-sustainable autonomy of battery-free soft actuators is derived from the self-sensing and feedback-controlled systems that perform long-term tasks. Actuators can interact with other functional components for effective and intelligent applications, including agricultural seeding, self-navigation of unstructured terrain, light modulation, power generation, and cargo transportation.

**Fig. 6:**
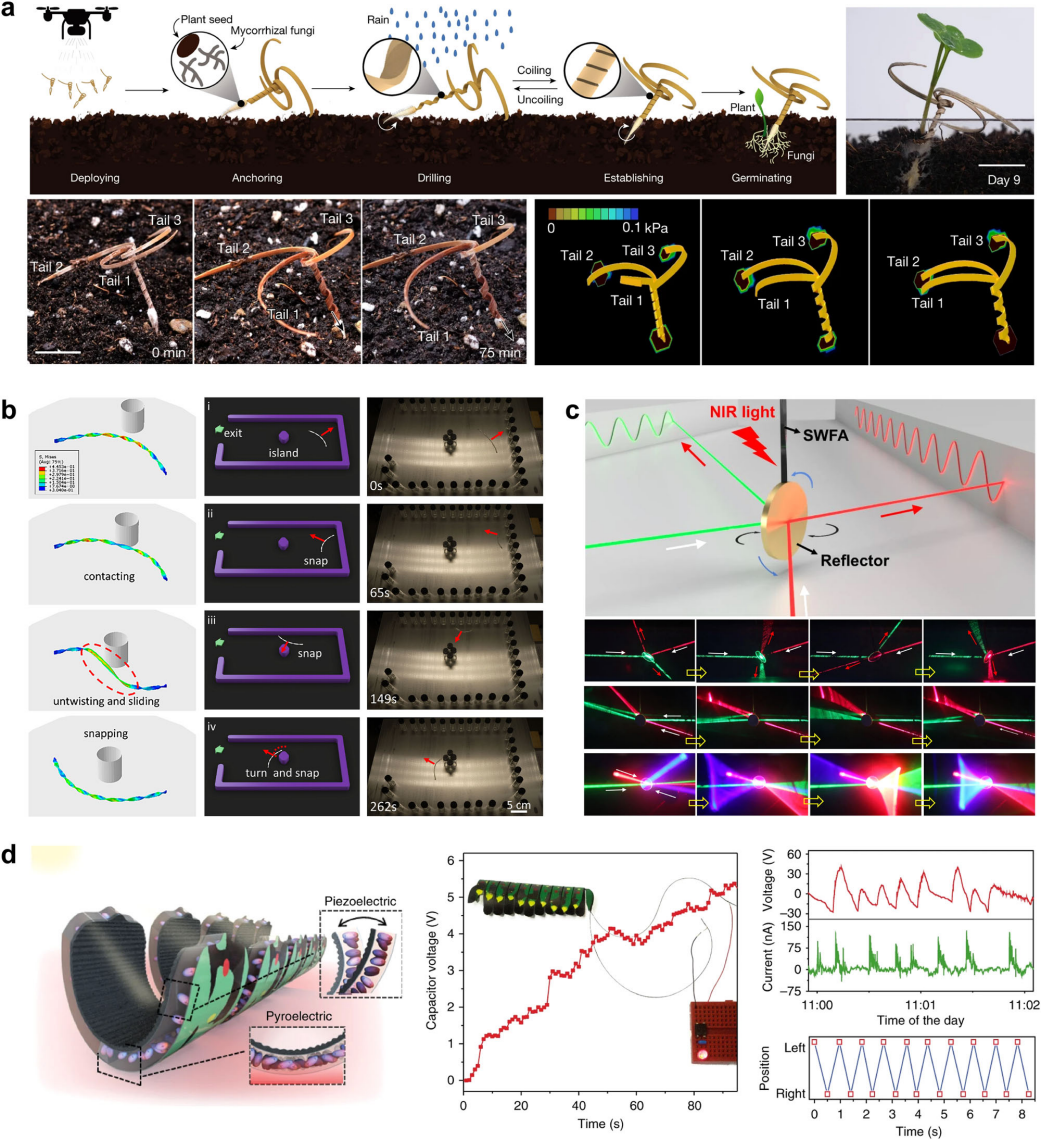
Self-sustainable morphing-induced applications of soft actuators. **a** Self-burying wooden robotic for sowing seeds. Reprinted with permission from Ref. ^90^, Springer Nature. **b** Adaptive navigation and physical intelligence in unstructured environments. Reprinted with permission from Ref. ^44^, PNAS. **c** Self-oscillatory laser modulation system. Reprinted with permission from Ref. ^79^, Springer Nature. **d** Self-propelled electricity generation. Reprinted with permission from Ref. ^86^, Springer Nature.

The self-drilling potential of natural seeds inspired the design of a wooden actuator capable of burying itself in the soil when exposed to rainfall (Fig. 6a). Robotic seed carriers have a spiral body with seed tips at each end^90,91^. The three tails at the other end were used to effectively anchor the seed in the soil and generate large rotational and thrust forces. The wooden actuator exposed to water could morph and unwind to realize the drilling action, and the corresponding drilling success rate reached 80%. Coupled with aerial seeding technology, carriers carry payloads of multiple seed sizes and contents and enhance germination rates. This study is an elegant example of an environmentally adaptive response and a specific task, highlighting the potential of actuator systems for autonomous operations. The embodied physical intelligence of soft actuators has been designed for self-adaptive movements in various unstructured environments^12,13,44,92,93^. As shown in Fig. 6b, the soft actuators were prepared by twisting and stretching the LCE strips along a straight centerline^44^. The thermal gradient drives helical actuators for self-rolling on various challenging substrates, such as sand ripples and rocks. More essentially, when facing obstacles, actuators can perform autonomous reversion because of spontaneous curvature changes and snap-throughs. Furthermore, soft robots can self-escape the confined spaces and complex obstacles. Physical intelligence is just like a “brain” embedded into actuator systems, enabling actuators to make decisions and regulate the motion modes by themselves. This study provides a facile approach for the design of highly intelligent systems. As illustrated in Fig. 6c, the modulation of the laser beams was achieved using a reconfigured twisted self-oscillating LCE ribbon^79^. In the laser-steering system, the self-oscillating ribbon acts as an actuator, the mirror reflector is regarded as a modulator, and a near-infrared light pointer is used as the constant stimulus. The modulation implements the 1D scanning with horizontal *φ* and vertical *δ* angular tuning range of about 360° and about 35° and 2D scanning, respectively. This self-oscillatory system has the advantages of light controllability, high efficiency, and a wide adjustment range.

Mechanical-electrical energy conversion is an interesting concept and a potential application of self-sustainable actuators, including mechano-electrical transduction, soft energy robotics, and energy-harvesting technologies. In recent years, scientists have developed three power generation mechanisms for autonomous actuator systems. The self-oscillatory generator was based on Faraday’s law of electromagnetic induction^34,94^. The light-triggered autonomous pendulum allows the coils to pass through an external magnetic field. The change in the magnetic flux contributes to the generation of induction signals, including voltage and current. The corresponding voltage is approximately 1 mV. A pair of pyro-/piezoelectric mechanistic intersections with self-oscillatory motion is shown in Fig. 6d. Thermo-mechano-electrical transduction is derived from the pyro/piezoelectric effect^86^. The open-circuit *V*_oc_ and *I*_sc_ signals reached approximately 45 V and 135 nA, respectively. The soft generator charged an external storage capacitor of 5 V and successfully lit a red LED. The third mechanism is based on a triboelectric nanogenerator (TENG). For the TENG systems, an auto-sustained analogous-triangle prototype actuator was placed on a PI/Al bi-layered triboelectric substrate^59^. This self-propelled motion led to repeated triboelectric charging and electrostatic induction. The maximum open-circuit voltage and frequency are approximately 400 mV and 1.4 Hz, respectively. Autonomous, sustainable morphing-induced actuators also have a few other functionalities and applications, including the transport and lifting of miniature cargos^95^, synchronized flapping wings^84^, and fluid flow motions^96^.

### Summary and outlook

Self-sustainable autonomous soft actuators serve as the evolutionary intelligence of stimulus-responsive materials, endowing shape-morphing materials with life-like perception, feedback, decision-making, and self-sustainable capabilities. In this perspective article, we summarize the recent advances in self-sustainable autonomous soft actuators. Raw shape-morphing materials mainly include LCEs/LCNs, bilayered materials with varied CTE, hydrogels, and some hygroscopic materials. Autonomous behaviors are divided into periodic self-oscillation and continuous self-rotation. Artificial self-sustainable mechanisms are out-of-equilibrium phenomena based on built-in negative feedback loops. The B-Z oscillating chemical reaction, light, thermal gradient, moisture gradient, and pressure are the five constant stimuli that are suitable for triggering self-sustaining locomotion. Autonomous concepts and modes include wave propagation, phototaxis, underwater motion, homeostatic oscillation, multimodal movement, and synchronization. Autonomously shape-morphing actuators have applications in agricultural seeding, self-navigation of unstructured terrain, light modulation, power generation, and cargo transportation.

Despite recent efforts and progress, several challenges remain unresolved. For stimulus-triggering methods, new stimulus-responsive modes should be created, such as electrical or magnetic-driven feedback-controlled systems, to improve maneuverability and expand application scenarios. To improve the motion modes and performance of autonomous actuators, novel modal locomotion methods, including jumping, climbing, and flying, must be embedded into self-sustaining systems^97^. These modes and functions require improvements in energy efficiency and performance, which rely on innovations in structures and materials. Many smart structures including bistable/multistable structures, topology structures, tensegrity structures, origami, and metamaterials can be embedded into soft robots to obtain novel mechanical properties, such as snapping motion and high degrees of freedom. In addition, scientists also need to develop the new reagents with high photothermal or electrothermal capabilities, molecular motors, artificial muscle materials and actuation mechanisms to achieve high frequency, large actuation force, and excellent loading capacity.

Higher-level intelligence systems for physical intelligence will be developed in the future. Actuators have complex bionic behavioral mechanisms and physical intelligence. Research efforts will no longer be limited to the learning of certain movement patterns. We need to establish complex feedback loops and responsive behavior mechanisms to impart “thoughts” or “cognition” to soft actuators. Regarding the application prospects, on the one hand, actuators are directly used in specific scenarios and autonomously interact with environmental changes, such as agriculture, thermal insulation/dissipation, room acoustics, and intelligent coating. On the other hand, autonomous actuators are considered as actuation components of soft robots for use in wireless smart machines that require no external power source. Furthermore, device integration has great potential to enable life-like functionality. Soft machines and robotics from micro/nano to meter-scale dimensions are expected to be used for intelligent perception and feedback of environmental changes, which will serve human-machine interactions, such as rescue missions and unstructured terrain exploration, wearables, biomedicine and rehabilitation. We hope that multiple disciplines (e.g., theoretical calculations, chemistry, materials, engineering, machinery, and agriculture) will undertake more collaborations to enrich the concepts, functions, and applications of self-sustainable autonomous actuation systems.
